# *N*-*tert*-Butyl *O*-2-isopropyl-5-methyl­cyclo­hexyl phenyl­phospho­namidate

**DOI:** 10.1107/S1600536811012864

**Published:** 2011-04-29

**Authors:** Li-Juan Liu, Fan-Jie Meng, Hao Xu, Daqi Wang, Chang-Qiu Zhao

**Affiliations:** a College of Chemistry and Chemical Engineering, Liaocheng University, Shandong 252059, People’s Republic of China

## Abstract

In the title compound, C_20_H_34_NO_2_P, the P atom has an irregular tetra­hedral environment and exhibits *S*_p_ chirality. In the crystal, weak inter­molecular N—H⋯O and C—H⋯O hydrogen bonds link the mol­ecules into chains extending in [010].

## Related literature

For the crystal structures of related P-chiral compounds, see: Chaloner *et al.* (1991 ▶); Meng *et al.* (2010 ▶).
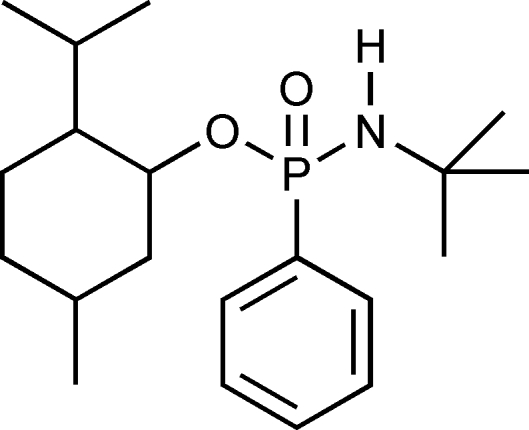


## Experimental

### 

#### Crystal data

C_20_H_34_NO_2_P
*M* *_r_* = 351.45
Orthorhombic, 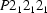

*a* = 8.305 (3) Å
*b* = 11.064 (4) Å
*c* = 22.557 (9) Å
*V* = 2072.8 (15) Å^3^
*Z* = 4
Mo *K*α radiation
μ = 0.14 mm^−1^
*T* = 298 K
0.45 × 0.40 × 0.37 mm

#### Data collection

Bruker SMART-1000 CCD area-detector diffractometer
Absorption correction: multi-scan (*SADABS*; Sheldrick, 1996 ▶) *T*_min_ = 0.938, *T*_max_ = 0.949
10336 measured reflections
3610 independent reflections
1993 reflections with *I* > 2σ(*I*)
*R*_int_ = 0.075

#### Refinement

*R*[*F* ^2^ > 2σ(*F* ^2^)] = 0.053
*wR*(*F* ^2^) = 0.130
*S* = 1.02
3610 reflections
223 parameters
114 restraints
H-atom parameters constrained
Δρ_max_ = 0.27 e Å^−3^
Δρ_min_ = −0.31 e Å^−3^
Absolute structure: Flack (1983 ▶), 2085 Friedel pairs
Flack parameter: 0.06 (17)

### 

Data collection: *SMART* (Bruker, 2007 ▶); cell refinement: *SAINT* (Bruker, 2007 ▶); data reduction: *SAINT*; program(s) used to solve structure: *SHELXS97* (Sheldrick, 2008 ▶); program(s) used to refine structure: *SHELXL97* (Sheldrick, 2008 ▶); molecular graphics: *SHELXTL* (Sheldrick, 2008 ▶); software used to prepare material for publication: *SHELXTL*.

## Supplementary Material

Crystal structure: contains datablocks I, global. DOI: 10.1107/S1600536811012864/cv5071sup1.cif

Structure factors: contains datablocks I. DOI: 10.1107/S1600536811012864/cv5071Isup2.hkl

Additional supplementary materials:  crystallographic information; 3D view; checkCIF report

## Figures and Tables

**Table 1:** Hydrogen-bond geometry (Å, °)

*D*—H⋯*A*	*D*—H	H⋯*A*	*D*⋯*A*	*D*—H⋯*A*
N1—H1⋯O2^i^	0.86	2.52	3.326 (4)	156
C13—H13⋯O2^i^	0.93	2.51	3.391 (5)	157

Symmetry code: (i) 
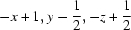
.

**Fig. 1.**
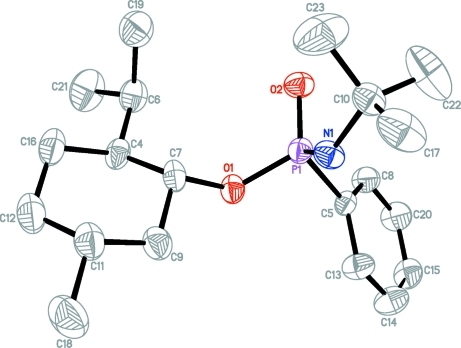
The molecular structure of (I) showing the atomic numbering and 30% probability displacement ellipsoids. H atoms have been omitted for clarity.

## supplementary crystallographic information

### Comment

We recently reported the crystal stucture of 2-isopropyl-5-methylcyclohexyl *N*-cyclohexyl-*P*- phenylphosphonamidate synthesized by the reaction of (*R*_p_)-*O*-menthyl phenylphosphinate with cyclohexylamine (Meng *et al.*, 2010). Herein we report the title compound (I) obtained by the reaction of the same phosphinate with *tert*-butylamine.

In (I) (Fig.1), the configuration of the central P atom was detemined as *S* and the four groups around the P atom form an irregular tetrahedron. A stable chair conformation was observed for the 2-isopropyl-5-methylcyclohexyloxy, in which the isopropyl, methyl and oxygen atom locate at equatorial bond. The absolute configuration of C_4_, C_7_, and C_11_ are *S*, *R*, and *R*, respectively. The bond angle around the P atom are normal and comparable with those observed in the related compounds (Meng *et al.*, 2010; Chaloner *et al.* 1991).

In the crystal structure, the molecules are linked by weak intermolecular N1—H1···O2 and C13—H13···O2 hodrogen bonds (Tabel 1) into chains extended in [010].

### Experimental

Carbon tetrachloride was added to a solution of (*R*_p_)-*O*-menthyl-phenylphosphonothioate dissolved in dry ether and *tert*-butylamine.The reaction mixture was stirred for 30 h at room temperature. The crystal suitable for X-ray diffraction was obtained by recrystallization with dichloromethane/hexane.

### Refinement

All H atoms were fixed geometrically (C—H = 0.93 - 0.98 Å; N—H = 0.86 Å), and treated as riding, with *U*_iso_(H) = 1.2-1.5 *U*_eq_of the parent atom.

### Crystal data

**Table:**

C_20_H_34_NO_2_P	*F*(000) = 768
*M**_r_* = 351.45	*D*_x_ = 1.126 Mg m^−^^3^
Orthorhombic, *P*2_1_2_1_2_1_	Mo *K*α radiation, λ = 0.71073 Å
Hall symbol: P 2ac 2ab	Cell parameters from 1474 reflections
*a* = 8.305 (3) Å	θ = 2.6–18.0°
*b* = 11.064 (4) Å	µ = 0.14 mm^−^^1^
*c* = 22.557 (9) Å	*T* = 298 K
*V* = 2072.8 (15) Å^3^	Block, colourless
*Z* = 4	0.45 × 0.40 × 0.37 mm

### Data collection

**Table:**

Bruker SMART-1000 CCD area-detector diffractometer	3610 independent reflections
Radiation source: fine-focus sealed tube	1993 reflections with *I* > 2σ(*I*)
graphite	*R*_int_ = 0.075
φ and ω scans	θ_max_ = 25.0°, θ_min_ = 2.6°
Absorption correction: multi-scan (*SADABS*; Sheldrick, 1996)	*h* = −9→9
*T*_min_ = 0.938, *T*_max_ = 0.949	*k* = −10→13
10336 measured reflections	*l* = −26→25

### Refinement

**Table:**

Refinement on *F*^2^	Secondary atom site location: difference Fourier map
Least-squares matrix: full	Hydrogen site location: inferred from neighbouring sites
*R*[*F*^2^ > 2σ(*F*^2^)] = 0.053	H-atom parameters constrained
*wR*(*F*^2^) = 0.130	*w* = 1/[σ^2^(*F*_o_^2^) + (0.0457*P*)^2^] where *P* = (*F*_o_^2^ + 2*F*_c_^2^)/3
*S* = 1.02	(Δ/σ)_max_ = 0.001
3610 reflections	Δρ_max_ = 0.27 e Å^−^^3^
223 parameters	Δρ_min_ = −0.31 e Å^−^^3^
114 restraints	Absolute structure: Flack (1983), 2085 Friedel pairs
Primary atom site location: structure-invariant direct methods	Flack parameter: 0.06 (17)

### Special details

**Table:**

Geometry. All e.s.d.'s (except the e.s.d. in the dihedral angle between two l.s. planes) are estimated using the full covariance matrix. The cell e.s.d.'s are taken into account individually in the estimation of e.s.d.'s in distances, angles and torsion angles; correlations between e.s.d.'s in cell parameters are only used when they are defined by crystal symmetry. An approximate (isotropic) treatment of cell e.s.d.'s is used for estimating e.s.d.'s involving l.s. planes.
Refinement. Refinement of *F*^2^ against ALL reflections. The weighted *R*-factor *wR* and goodness of fit *S* are based on *F*^2^, conventional *R*-factors *R* are based on *F*, with *F* set to zero for negative *F*^2^. The threshold expression of *F*^2^ > σ(*F*^2^) is used only for calculating *R*-factors(gt) *etc*. and is not relevant to the choice of reflections for refinement. *R*-factors based on *F*^2^ are statistically about twice as large as those based on *F*, and *R*- factors based on ALL data will be even larger.

### Fractional atomic coordinates and isotropic or equivalent isotropic displacement parameters (Å^2^)

**Table:**

	*x*	*y*	*z*	*U*_iso_*/*U*_eq_	
P1	0.54415 (14)	0.34918 (10)	0.23972 (5)	0.0462 (3)	
O1	0.6726 (3)	0.2945 (3)	0.19552 (12)	0.0503 (8)	
O2	0.5009 (3)	0.4756 (2)	0.23040 (13)	0.0578 (9)	
N1	0.3903 (4)	0.2570 (3)	0.23664 (16)	0.0521 (9)	
H1	0.4139	0.1816	0.2332	0.063*	
C4	0.7421 (5)	0.3383 (5)	0.09282 (17)	0.0586 (13)	
H4	0.8542	0.3192	0.1027	0.070*	
C5	0.6512 (5)	0.3284 (4)	0.30808 (17)	0.0418 (11)	
C6	0.7238 (7)	0.4734 (5)	0.1000 (2)	0.0679 (14)	
H6	0.7301	0.4912	0.1425	0.082*	
C7	0.6383 (5)	0.2641 (4)	0.13399 (18)	0.0511 (12)	
H7	0.5247	0.2806	0.1256	0.061*	
C8	0.6923 (6)	0.4277 (4)	0.3410 (2)	0.0624 (14)	
H8	0.6632	0.5044	0.3281	0.075*	
C9	0.6699 (6)	0.1301 (4)	0.1270 (2)	0.0636 (14)	
H9A	0.7795	0.1132	0.1394	0.076*	
H9B	0.5983	0.0861	0.1533	0.076*	
C10	0.2175 (5)	0.2851 (4)	0.2390 (2)	0.0605 (12)	
C11	0.6471 (7)	0.0842 (5)	0.0651 (2)	0.0708 (15)	
H11	0.5336	0.0970	0.0551	0.085*	
C12	0.7434 (7)	0.1585 (6)	0.0229 (2)	0.0831 (17)	
H12A	0.7165	0.1348	−0.0173	0.100*	
H12B	0.8568	0.1418	0.0291	0.100*	
C13	0.6942 (6)	0.2165 (4)	0.3286 (2)	0.0627 (14)	
H13	0.6688	0.1482	0.3065	0.075*	
C14	0.7741 (6)	0.2041 (5)	0.3811 (2)	0.0750 (16)	
H14	0.7992	0.1275	0.3952	0.090*	
C15	0.8169 (6)	0.3034 (6)	0.4129 (2)	0.0723 (16)	
H15	0.8739	0.2941	0.4481	0.087*	
C16	0.7145 (6)	0.2930 (5)	0.0299 (2)	0.0732 (16)	
H16A	0.6046	0.3111	0.0183	0.088*	
H16B	0.7856	0.3362	0.0032	0.088*	
C17	0.1234 (6)	0.1719 (5)	0.2447 (3)	0.1004 (18)	
H17A	0.1550	0.1304	0.2802	0.151*	
H17B	0.1435	0.1211	0.2110	0.151*	
H17C	0.0107	0.1908	0.2467	0.151*	
C18	0.6792 (8)	−0.0509 (5)	0.0587 (2)	0.103 (2)	
H18A	0.6648	−0.0744	0.0181	0.154*	
H18B	0.6054	−0.0951	0.0833	0.154*	
H18C	0.7876	−0.0683	0.0708	0.154*	
C19	0.5637 (7)	0.5245 (5)	0.0774 (2)	0.0915 (19)	
H19A	0.5523	0.5065	0.0360	0.137*	
H19B	0.5621	0.6105	0.0830	0.137*	
H19C	0.4764	0.4885	0.0990	0.137*	
C20	0.7781 (7)	0.4143 (5)	0.3941 (2)	0.0723 (16)	
H20	0.8077	0.4819	0.4160	0.087*	
C21	0.8611 (8)	0.5424 (6)	0.0696 (3)	0.109 (2)	
H21A	0.9626	0.5089	0.0817	0.163*	
H21B	0.8566	0.6261	0.0807	0.163*	
H21C	0.8502	0.5354	0.0274	0.163*	
C22	0.1788 (8)	0.3622 (6)	0.2900 (3)	0.136 (2)	
H22A	0.0641	0.3665	0.2948	0.204*	
H22B	0.2210	0.4419	0.2835	0.204*	
H22C	0.2260	0.3286	0.3252	0.204*	
C23	0.1671 (8)	0.3533 (7)	0.1851 (3)	0.137 (2)	
H23A	0.0565	0.3770	0.1890	0.206*	
H23B	0.1793	0.3028	0.1508	0.206*	
H23C	0.2332	0.4240	0.1809	0.206*	

### Atomic displacement parameters (Å^2^)

**Table:**

	*U*^11^	*U*^22^	*U*^33^	*U*^12^	*U*^13^	*U*^23^
P1	0.0430 (6)	0.0520 (7)	0.0436 (7)	−0.0011 (6)	−0.0006 (6)	0.0021 (6)
O1	0.0398 (17)	0.071 (2)	0.0398 (17)	−0.0001 (16)	−0.0011 (14)	−0.0013 (14)
O2	0.054 (2)	0.0493 (18)	0.070 (2)	−0.0005 (15)	−0.0028 (16)	0.0074 (15)
N1	0.037 (2)	0.052 (2)	0.068 (2)	−0.0012 (17)	0.0005 (19)	−0.0009 (19)
C4	0.042 (3)	0.093 (4)	0.041 (3)	0.000 (3)	−0.004 (2)	0.006 (3)
C5	0.040 (3)	0.051 (3)	0.035 (2)	−0.003 (2)	−0.0010 (19)	0.005 (2)
C6	0.067 (4)	0.075 (4)	0.062 (3)	−0.014 (3)	0.004 (3)	0.001 (3)
C7	0.040 (3)	0.076 (4)	0.038 (3)	−0.002 (3)	0.003 (2)	−0.009 (2)
C8	0.077 (4)	0.057 (3)	0.053 (3)	0.008 (3)	−0.009 (3)	−0.005 (3)
C9	0.053 (3)	0.080 (4)	0.058 (3)	0.005 (3)	0.003 (2)	−0.005 (3)
C10	0.042 (3)	0.065 (3)	0.074 (3)	−0.004 (2)	0.001 (3)	−0.003 (3)
C11	0.065 (4)	0.090 (4)	0.057 (3)	0.004 (3)	0.001 (3)	−0.011 (3)
C12	0.078 (4)	0.115 (5)	0.056 (3)	0.008 (4)	0.008 (3)	−0.014 (3)
C13	0.074 (4)	0.050 (3)	0.064 (3)	0.001 (3)	−0.017 (3)	−0.002 (2)
C14	0.076 (4)	0.076 (4)	0.073 (4)	−0.001 (3)	−0.028 (3)	0.010 (3)
C15	0.067 (4)	0.101 (5)	0.048 (3)	0.004 (3)	−0.019 (3)	0.002 (3)
C16	0.072 (4)	0.104 (5)	0.044 (3)	−0.003 (3)	0.002 (3)	0.001 (3)
C17	0.055 (3)	0.079 (4)	0.168 (5)	−0.013 (3)	0.010 (4)	−0.014 (4)
C18	0.120 (6)	0.098 (5)	0.090 (4)	0.020 (4)	−0.006 (4)	−0.037 (3)
C19	0.097 (5)	0.084 (4)	0.093 (4)	−0.006 (4)	0.017 (4)	0.014 (3)
C20	0.093 (5)	0.064 (4)	0.059 (4)	−0.002 (4)	−0.018 (3)	−0.016 (3)
C21	0.098 (5)	0.118 (5)	0.110 (5)	−0.046 (4)	0.018 (4)	0.020 (4)
C22	0.081 (4)	0.143 (5)	0.183 (6)	−0.013 (4)	0.033 (4)	−0.072 (5)
C23	0.074 (4)	0.166 (6)	0.172 (6)	−0.017 (4)	−0.036 (4)	0.072 (5)

### Geometric parameters (Å, °)

**Table:**

P1—O2	1.459 (3)	C12—H12A	0.9700
P1—O1	1.581 (3)	C12—H12B	0.9700
P1—N1	1.636 (3)	C13—C14	1.365 (6)
P1—C5	1.794 (4)	C13—H13	0.9300
O1—C7	1.456 (5)	C14—C15	1.359 (6)
N1—C10	1.470 (5)	C14—H14	0.9300
N1—H1	0.8600	C15—C20	1.338 (6)
C4—C7	1.510 (6)	C15—H15	0.9300
C4—C6	1.512 (6)	C16—H16A	0.9700
C4—C16	1.524 (6)	C16—H16B	0.9700
C4—H4	0.9800	C17—H17A	0.9600
C5—C13	1.369 (6)	C17—H17B	0.9600
C5—C8	1.370 (6)	C17—H17C	0.9600
C6—C19	1.533 (7)	C18—H18A	0.9600
C6—C21	1.534 (7)	C18—H18B	0.9600
C6—H6	0.9800	C18—H18C	0.9600
C7—C9	1.514 (6)	C19—H19A	0.9600
C7—H7	0.9800	C19—H19B	0.9600
C8—C20	1.400 (6)	C19—H19C	0.9600
C8—H8	0.9300	C20—H20	0.9300
C9—C11	1.497 (6)	C21—H21A	0.9600
C9—H9A	0.9700	C21—H21B	0.9600
C9—H9B	0.9700	C21—H21C	0.9600
C10—C22	1.469 (7)	C22—H22A	0.9600
C10—C17	1.483 (6)	C22—H22B	0.9600
C10—C23	1.490 (7)	C22—H22C	0.9600
C11—C12	1.490 (7)	C23—H23A	0.9600
C11—C18	1.525 (7)	C23—H23B	0.9600
C11—H11	0.9800	C23—H23C	0.9600
C12—C16	1.516 (7)		
			
O2—P1—O1	116.25 (17)	C16—C12—H12B	109.0
O2—P1—N1	113.53 (18)	H12A—C12—H12B	107.8
O1—P1—N1	105.14 (17)	C5—C13—C14	120.7 (4)
O2—P1—C5	111.6 (2)	C5—C13—H13	119.6
O1—P1—C5	99.14 (18)	C14—C13—H13	119.6
N1—P1—C5	110.08 (19)	C15—C14—C13	120.3 (5)
C7—O1—P1	123.9 (3)	C15—C14—H14	119.9
C10—N1—P1	129.0 (3)	C13—C14—H14	119.9
C10—N1—H1	115.5	C20—C15—C14	120.7 (5)
P1—N1—H1	115.5	C20—C15—H15	119.7
C7—C4—C6	114.5 (4)	C14—C15—H15	119.7
C7—C4—C16	108.0 (4)	C12—C16—C4	113.3 (4)
C6—C4—C16	114.2 (4)	C12—C16—H16A	108.9
C7—C4—H4	106.5	C4—C16—H16A	108.9
C6—C4—H4	106.5	C12—C16—H16B	108.9
C16—C4—H4	106.5	C4—C16—H16B	108.9
C13—C5—C8	118.5 (4)	H16A—C16—H16B	107.7
C13—C5—P1	122.4 (4)	C10—C17—H17A	109.5
C8—C5—P1	119.2 (4)	C10—C17—H17B	109.5
C4—C6—C19	114.6 (4)	H17A—C17—H17B	109.5
C4—C6—C21	111.6 (5)	C10—C17—H17C	109.5
C19—C6—C21	108.2 (4)	H17A—C17—H17C	109.5
C4—C6—H6	107.4	H17B—C17—H17C	109.5
C19—C6—H6	107.4	C11—C18—H18A	109.5
C21—C6—H6	107.4	C11—C18—H18B	109.5
O1—C7—C4	110.4 (4)	H18A—C18—H18B	109.5
O1—C7—C9	107.0 (3)	C11—C18—H18C	109.5
C4—C7—C9	111.7 (4)	H18A—C18—H18C	109.5
O1—C7—H7	109.2	H18B—C18—H18C	109.5
C4—C7—H7	109.2	C6—C19—H19A	109.5
C9—C7—H7	109.2	C6—C19—H19B	109.5
C5—C8—C20	120.4 (5)	H19A—C19—H19B	109.5
C5—C8—H8	119.8	C6—C19—H19C	109.5
C20—C8—H8	119.8	H19A—C19—H19C	109.5
C11—C9—C7	114.0 (4)	H19B—C19—H19C	109.5
C11—C9—H9A	108.7	C15—C20—C8	119.4 (5)
C7—C9—H9A	108.7	C15—C20—H20	120.3
C11—C9—H9B	108.7	C8—C20—H20	120.3
C7—C9—H9B	108.7	C6—C21—H21A	109.5
H9A—C9—H9B	107.6	C6—C21—H21B	109.5
N1—C10—C22	111.4 (4)	H21A—C21—H21B	109.5
N1—C10—C17	109.8 (4)	C6—C21—H21C	109.5
C22—C10—C17	107.8 (5)	H21A—C21—H21C	109.5
N1—C10—C23	110.6 (4)	H21B—C21—H21C	109.5
C22—C10—C23	106.5 (5)	C10—C22—H22A	109.5
C17—C10—C23	110.6 (5)	C10—C22—H22B	109.5
C12—C11—C9	109.9 (4)	H22A—C22—H22B	109.5
C12—C11—C18	112.7 (5)	C10—C22—H22C	109.5
C9—C11—C18	113.5 (4)	H22A—C22—H22C	109.5
C12—C11—H11	106.8	H22B—C22—H22C	109.5
C9—C11—H11	106.8	C10—C23—H23A	109.5
C18—C11—H11	106.8	C10—C23—H23B	109.5
C11—C12—C16	113.0 (4)	H23A—C23—H23B	109.5
C11—C12—H12A	109.0	C10—C23—H23C	109.5
C16—C12—H12A	109.0	H23A—C23—H23C	109.5
C11—C12—H12B	109.0	H23B—C23—H23C	109.5
			
O2—P1—O1—C7	−73.5 (3)	C16—C4—C7—C9	55.0 (5)
N1—P1—O1—C7	52.9 (3)	C13—C5—C8—C20	0.7 (7)
C5—P1—O1—C7	166.8 (3)	P1—C5—C8—C20	−179.0 (4)
O2—P1—N1—C10	−14.7 (5)	O1—C7—C9—C11	−177.5 (4)
O1—P1—N1—C10	−142.9 (4)	C4—C7—C9—C11	−56.6 (5)
C5—P1—N1—C10	111.2 (4)	P1—N1—C10—C22	−51.2 (6)
O2—P1—C5—C13	175.2 (4)	P1—N1—C10—C17	−170.6 (4)
O1—P1—C5—C13	−61.8 (4)	P1—N1—C10—C23	67.1 (6)
N1—P1—C5—C13	48.1 (4)	C7—C9—C11—C12	52.8 (6)
O2—P1—C5—C8	−5.2 (4)	C7—C9—C11—C18	−180.0 (5)
O1—P1—C5—C8	117.9 (4)	C9—C11—C12—C16	−51.2 (6)
N1—P1—C5—C8	−132.2 (3)	C18—C11—C12—C16	−178.9 (4)
C7—C4—C6—C19	−71.5 (5)	C8—C5—C13—C14	1.0 (7)
C16—C4—C6—C19	53.6 (6)	P1—C5—C13—C14	−179.3 (4)
C7—C4—C6—C21	165.0 (4)	C5—C13—C14—C15	−2.3 (8)
C16—C4—C6—C21	−69.8 (6)	C13—C14—C15—C20	1.7 (9)
P1—O1—C7—C4	118.4 (4)	C11—C12—C16—C4	54.8 (6)
P1—O1—C7—C9	−119.8 (3)	C7—C4—C16—C12	−54.9 (6)
C6—C4—C7—O1	−57.7 (5)	C6—C4—C16—C12	176.5 (4)
C16—C4—C7—O1	173.9 (4)	C14—C15—C20—C8	0.0 (9)
C6—C4—C7—C9	−176.6 (4)	C5—C8—C20—C15	−1.2 (8)

### Hydrogen-bond geometry (Å, °)

**Table:**

*D*—H···*A*	*D*—H	H···*A*	*D*···*A*	*D*—H···*A*
N1—H1···O2^i^	0.86	2.52	3.326 (4)	156
C13—H13···O2^i^	0.93	2.51	3.391 (5)	157

Symmetry codes: (i) −*x*+1, *y*−1/2, −*z*+1/2.
